# In vitro assessment of skin sensitization, irritability and toxicity of bacteriocins and reuterin for possible topical applications

**DOI:** 10.1038/s41598-022-08441-4

**Published:** 2022-03-17

**Authors:** Samira Soltani, Yvan Boutin, Frédéric Couture, Eric Biron, Muriel Subirade, Ismail Fliss

**Affiliations:** 1 Food Science Department, Food and Agriculture Faculty, Laval University, Rue de l’Agriculture, Local 1312A, Quebec, Canada; 2 TransBIOTech, 201 Rue Mgr Bourget, Lévis, QC G6V 6Z9 Canada; 3 Faculty of Pharmacy, Laval University and Laboratory of Medicinal Chemistry, CHU de Québec Research Center, Quebec, Canada; 4 Institute of Nutrition and Functional Foods, Laval University, Quebec, QC Canada

**Keywords:** Microbiology, Antimicrobials, Bacteria, Toxicology

## Abstract

Bacteriocins and reuterin are promising antimicrobials for application in food, veterinary, and medical sectors. In the light of their high potential for application in hand sanitizer, we investigated the skin toxicity of reuterin, microcin J25, pediocin PA-1, bactofencin A, and nisin Z in vitro using neutral red and LDH release assays on NHEK cells. We determined their skin sensitization potential using the human cell line activation test (h-CLAT). Their skin irritation potential was measured on human epidermal model EpiDerm™. We showed that the viability and membrane integrity of NHEK cells remained unaltered after exposure to bacteriocins and reuterin at concentrations up to 400 µg/mL and 80 mg/mL, respectively. Furthermore, microcin J25 and reuterin showed no skin sensitization at concentrations up to 100 µg/mL and 40 mg/mL, respectively, while pediocin PA-1, bactofencin A, and nisin Z caused sensitization at concentrations higher than 100 µg/mL. Tissue viability was unaffected in presence of bacteriocins and reuterin at concentrations up to 200 µg/mL and 40 mg/mL, respectively, which was confirmed by measuring cytokine IL-1α and IL-8 levels and by histological analysis. In conclusion, the current study provides scientific evidence that some bacteriocins and reuterin, could be safely applied topically as sanitizers at recommended concentrations.

## Introduction

Sanitation and disinfection programs are crucial to combat bacterial and viral contamination in the food, medical, and veterinary sectors. The hands of workers must be sanitized or disinfected along with the surfaces and equipment. Hands sanitizers can be of two types: alcohol-based (ABHS) and non-alcohol-based (NABHS). ABHS contains one or more types of alcohols, such as ethanol, isopropanol, or *n*-propanol, while antiseptic chemical compounds are the active ingredients in NABHS, including quaternary ammonium, chlorohexidine, triclosan, and iodine^1,2^. Alcohol-based sanitizers containing 60–90% alcohol are the most effective and commonly used in the health sector because of their broad spectrum of activity and rapid action^3^. However, they are flammable and toxic if ingested^4^. Excessive use of sanitizers containing alcohol and hydrogen peroxide dries and damages the skin, making it more susceptible to indirect infection through skin disorders^5^. In addition, frequent use of alcohol and hydrogen peroxide might make microorganisms resistant to them^6^. On the other hand, the use of chemical compounds in NABHS incites concerns over their safety and toxicity. For instance, triclosan is harmful to humans and the ecosystem, and the Food and Drug Administration (FDA) has banned its use in antiseptic products since September 2016^7^. Benzalkonium chloride, expected to replace triclosan, showed acute lethal toxicity at lower hundreds of µg/mL or lower mg/mL levels in model organisms^8^. Hence, we need to develop sanitizers, which has a high degree of antimicrobial efficacy while posing no risk of toxicity to humans. Some products as “Green” sanitizers have been developed based on natural antiseptic agents such as plant extracts, essential oil, coconut oil, extrudates, etc.^9^.

Many bacteria, including *Lactobacillus* species, produce a variety of antimicrobial substances such as bacteriocins, organic acids, hydrogen peroxide and many low molecular weight compounds such as fatty acids, and reuterin^10^. Bacteriocins are ribosomally-synthesized antimicrobial peptides produced by bacteria and can have a broad or narrow spectrum of inhibitory activity^11^. Bacteriocins are promising antimicrobial compounds with potential applications in food, veterinary, and clinical settings. They have been extensively studied as bio-preservatives to target food spoilage and pathogenic bacteria in dairy products, juice, and meat, etc.^12^. However, only nisin and pediocin are commercially available as preservatives. Nisin remains the only bacteriocin approved as a bio-preservative^13^. Interestingly, several studies have reported the inhibitory effects of bacteriocins against antibiotic-resistant strains^11^. Reuterin is a low molecular weight aldehyde produced by bioconversion of glycerol by the lactic acid bacteria (*Lactobacillus reuteri)*. The antimicrobial activity of reuterin was extensively studied^14–17^. We previously demonstrated that up to 20 mg/mL of reuterin could be safely used as a bio-preservative and therapeutic agent^18^.

Although reuterin and several bacteriocins are produced by GRAS bacteria, they still need to meet specific safety requirements to be legally approved and used on a routine basis for different food, medical and veterinary applications. Toxicity data are necessary to determine the physiological, biochemical, and toxicological effect of a test compound and predict its safe doses^19^. We recently reviewed the toxicity and safety of bacteriocins^20^ and observed a lack of studies on the issue. Moreover, currently there are no guidelines describing standard protocols for assessing bacteriocins toxicity. However, a few studies have addressed the oral toxicity of bacteriocins and reuterin^20–22^. To our knowledge, there are no studies on the dermal toxicity of bacteriocins or any other natural compounds produced by the *Lactobacillus* species. Testing for skin toxicity is essential in toxicological frameworks for any formulation, both topical and intradermal. Hence, we need safety data, including long-term toxicity tests, for hand sanitizers and disinfectants to protect consumers from side effects. Therefore, systematic data by conducting in vitro and in vivo tests for toxicity, allergenicity, and skin irritability must be provided.

The objective of the current study is thus to provide a complete portrait of dermal toxicity and safety of reuterin and selected bacteriocins namely; microcin J25, pediocin PA-1, nisin, bactofencin A, including their cytotoxicity, sensitization, and skin irritability based on in vitro tests. We adhered to the Organization for Economic Co-operation and Development (OECD) guidelines on in vitro testing of substances for topical application^23,24^. First, all compounds were produced and purified (> 95% purity) and their cytotoxicity was assessed using the neutral red and LDH release assays on normal human epidermal keratinocytes (NHEK) cells. A human cell line activation test (h-CLAT assay) was performed to determine their skin sensitization potential by measuring the expression levels of the CD54 and CD86 membrane markers in the human monocytic leukemia cell line (THP-1 cells). Finally, a three-dimensional EpiDerm™ culture (EPI-200) as a human skin equivalent was used to systematically study the effect of the selected bacteriocins and reuterin on tissue viability, structure, and release of proinflammatory cytokine secretion.

## Results

### Production and purification of bacteriocins and reuterin

**Figure 1:**
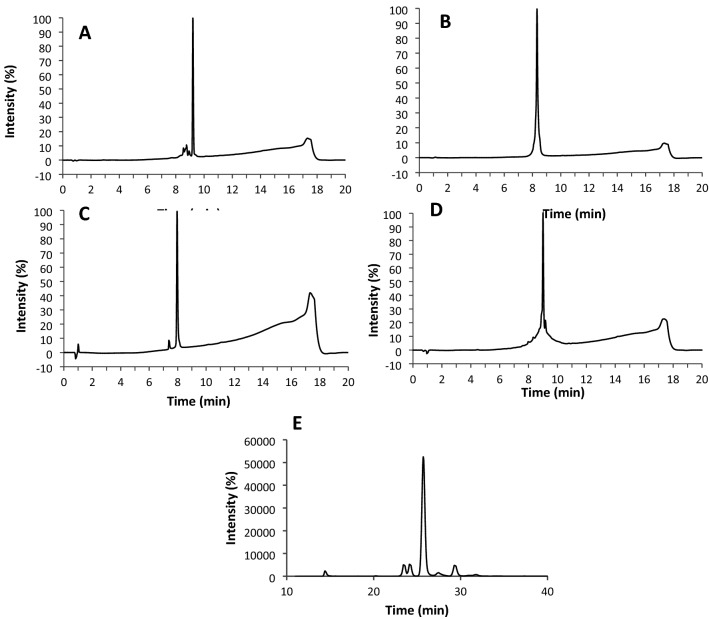
HPLC chromatogram of (**A**) microcin J25, (**B**) pediocin PA-1, (**C**) bactofencin A, (**D**) nisin Z, (**E**) reuterin.

The HPLC chromatogram, showed that microcin J25, pediocin PA-1, bactofencin A, nisin Z and reuterin were prepared with high purity (> 95%) (Fig. 1A–E). The identity and high purity were further confirmed by LC–MS analysis. For reuterin a high glycerol bioconversion rate was obtained, as shown by the negligible amount of residual glycerol (Fig. 1E).

**Figure 2:**
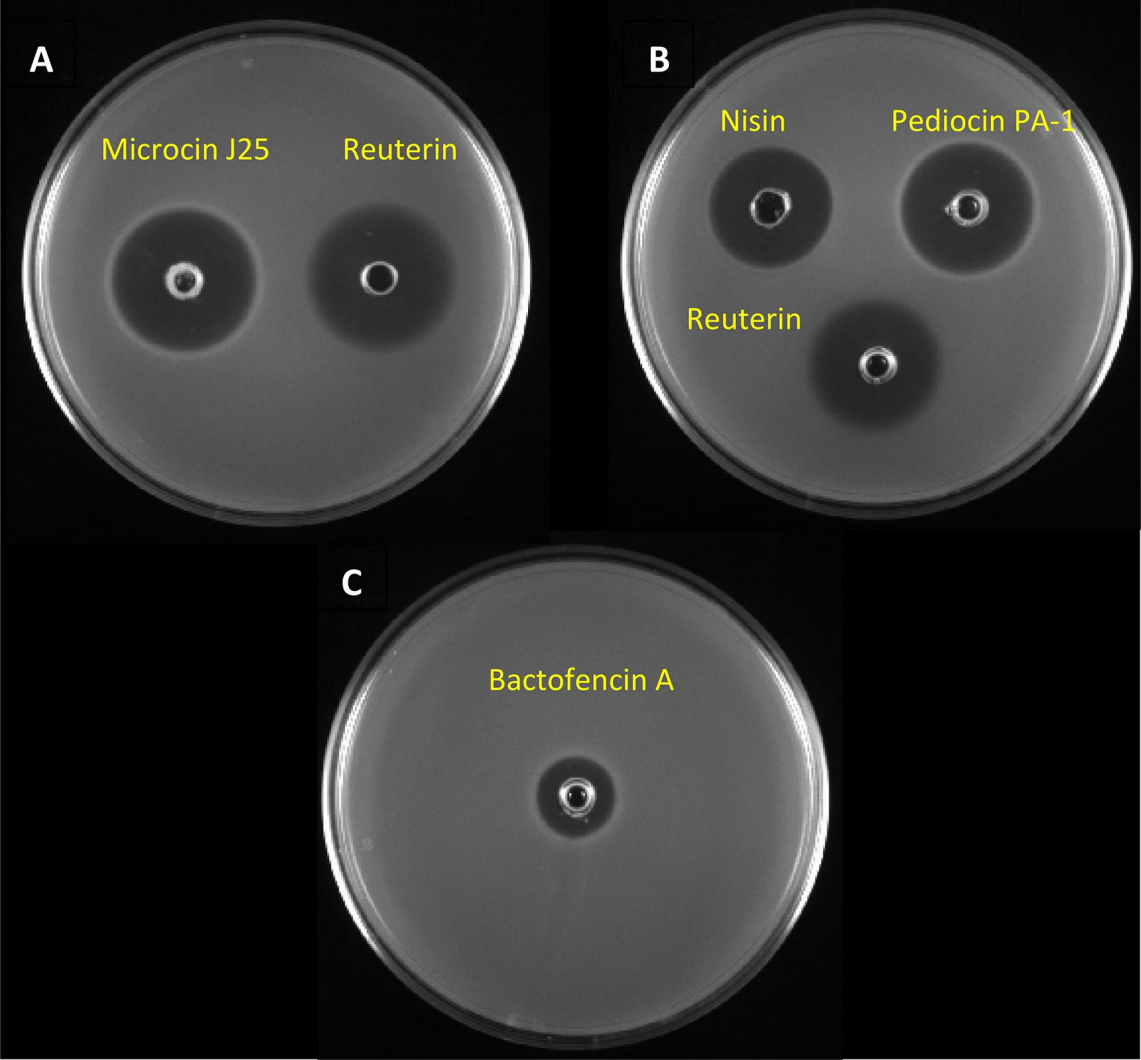
Inhibitory activity of bacteriocins and reuterin. (**A**) Microcin J25, 200 µg/mL, and reuterin 20 mg/mL against *S*. Newport ATCC 6962. (**B**) Pediocin PA-1 200 µg/mL and nisin Z 400 µg/mL, and reuterin 20 mg/mL against *L. ivanovii* HPB28. (**C**) Bactofencin A 500 µg/mL against *S. aureus* ATCC 6538.

The inhibitory activity of purified bacteriocins (microcin J25 200 µg/mL, pediocin PA-1 200 µg/mL, nisin Z 400 µg/mL, bactofencin A 500 µg/mL) and reuterin (20 mg/mL) was confirmed using an agar well diffusion assay (Fig. 2A–C). Microcin J25 and reuterin showed 26 mm and 25 mm inhibition zones against *Salmonella enterica* subsp. *enterica* serovar Newport ATCC 6962 (later referred to as S. Newport, ATCC 6962), respectively (Fig. 2A). Pediocin PA-1, nisin Z, and reuterin showed 22 mm, 19 mm, and 22 mm zones of inhibition against *Listeria ivanovii* HPB28, respectively (Fig. 2B). Bactofencin A inhibited *Staphylococcus aureus* with an inhibition zone of 13 mm (Fig. 2C).

### Neutral red assay

**Figure 3:**
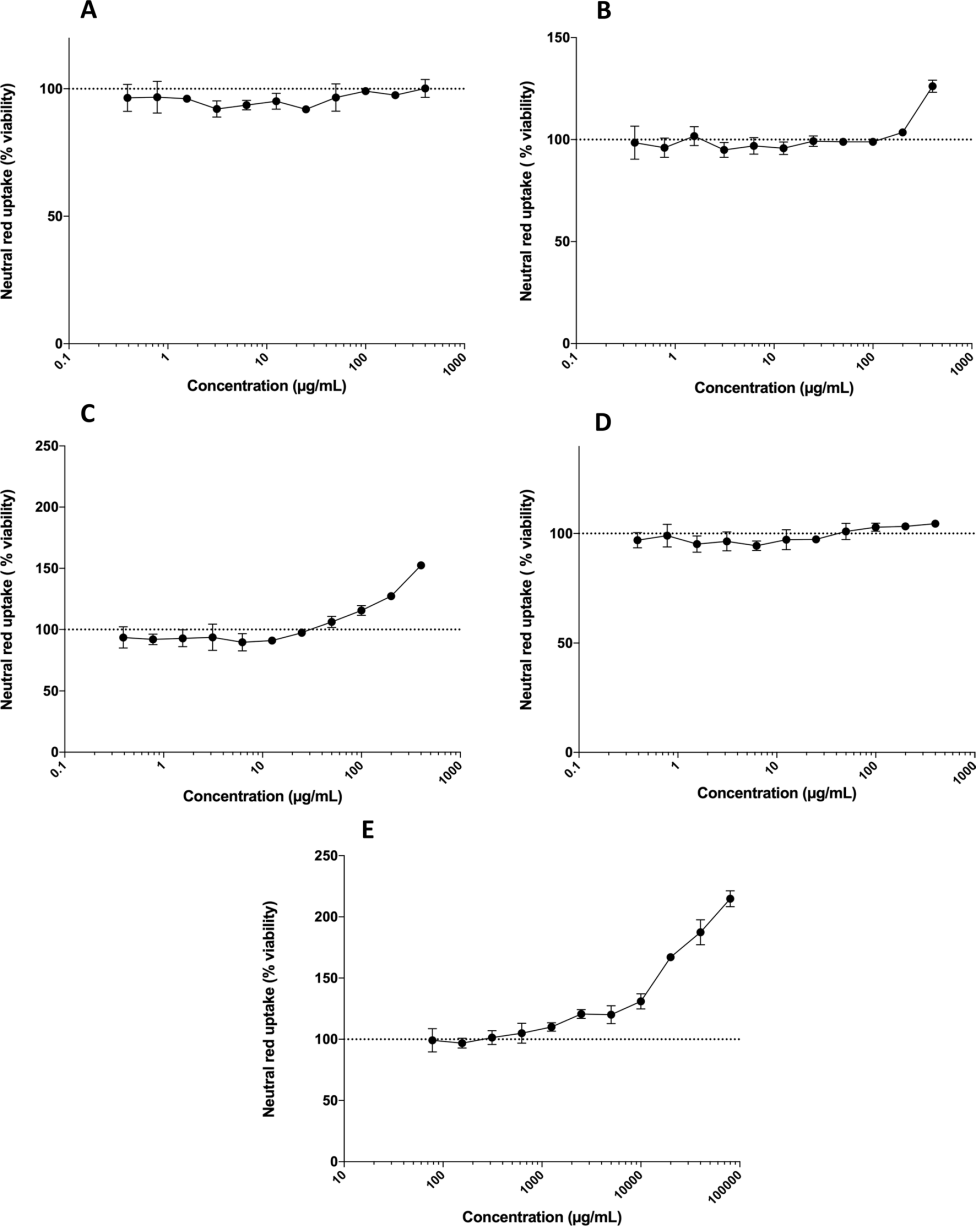
The effect of increasing concentrations of (**A**) MccJ25 (**B**) Pediocin PA-1 (**C**) Bactofencin A (**D**) Nisin, and (**E**) Reuterin on the viability of NHEK cells after 24 h of exposure using the neutral red assay. Viability is presented as a percentage of total cell viability. The control represents 100% viability. Concentration range for reuterin was 0.8–80 mg/mL and for other bacteriocins 0.4–400 μg/mL. Error bars show the standard error of mean (n = 3).

The cytotoxicity effects of increasing concentrations of microcin J25, pediocin PA-1, bactofencin A, nisin Z and reuterin on metabolic activity (reduction in lysosomal function) of NHEK cells were detected using neutral red assay after 24 h exposing them to the compounds (Fig. 3A–E). The results showed that bacteriocins and reuterin did not affect cell viability up to the highest concentration tested (400 µg/mL for bacteriocins and 80 mg/mL for reuterin). Interestingly, bactofencin A and reuterin increased the metabolic activity of the cells at concentrations up to 400 µg/mL and 80 mg/mL, respectively, in a dose-dependent manner that was 152% and 220% more than that of the controls, respectively (Fig. 3 C, E). Similarly, NHEK cell viability increased up to 126% upon exposure to 400 µg/mL of pediocin PA-1 (Fig. 3B).

### LDH release assay

**Figure 4:**
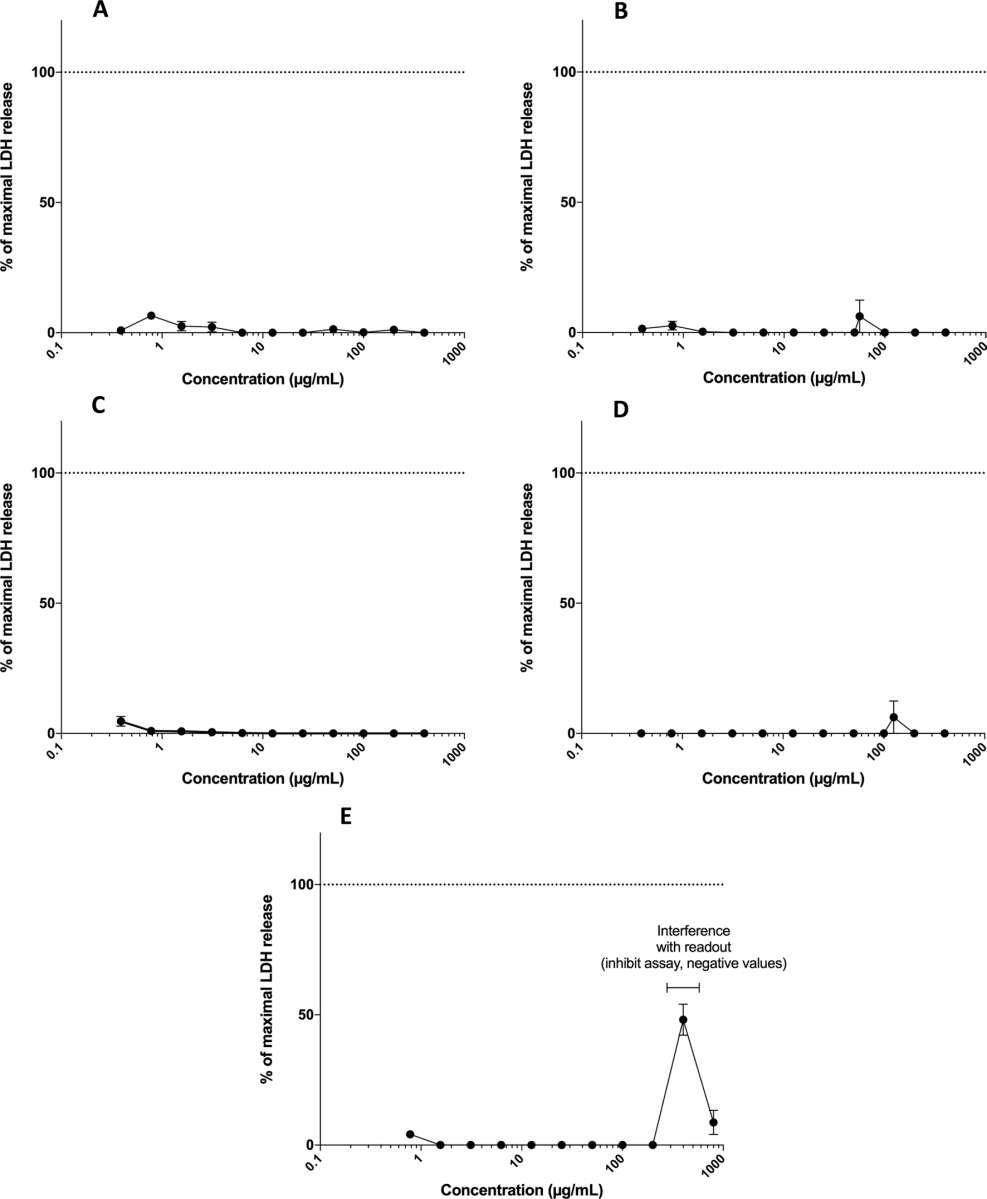
The effect of increasing concentration of (**A**) microcin J25, (**B**) pediocin PA-1, (**C**) bactofencin A, (**D**) nisin Z, and (**E**) reuterin on LDH release (in percentage) of NHEK cells after 24 h exposure. Lysis buffer, used as a positive control, represented 100% LDH release. Concentration range for bacteriocins was 0.4–400 µg/mL and for reuterin 0.8–80 mg/mL. Data are represented as means of three replicates, and error bars show the standard deviation of the mean (n = 3).

The effect of increasing concentrations of bacteriocins and reuterin on membrane integrity of NHEK cells was measured using the LDH release assay upon 24 h after initial exposure (Fig. 4). Microcin J25, pediocin PA-1, bactofencin A, nisin Z, and reuterin did not cause any concentration-dependent increase in extracellular LDH, indicating that membrane integrity was not affected up to a concentration of 400 µg/mL for bacteriocins and 80 mg/mL for reuterin (Fig. 4A–E). Reuterin, gave negative values for the assay at 40 mg/mL and 80 mg/mL, as it interfered with the readout (Fig. 4E).

### h-CLAT assay

The results of h-CLAT for bacteriocins and reuterin are outlined in Table 1. According to OECD guidelines^24^, a substance is considered positive and categorized as a sensitizer if two independent runs are positive for CD54 and/or CD86. In general, our results indicated that cell sensitization varied from one antimicrobial to another and depended on the final concentration of compounds. Pediocin PA-1 at 100 µg/mL was positive for both CD54 and CD86 (represented as RFI percentage). Bactofencin A and nisin Z were positive for both CD54 and CD86 at 200 µg/mL. However, when used at 100 µg/mL, only RFI for CD54 exceeded 200%, and was positive. Microcin J25 and reuterin were negative for both CD54 and CD86 at concentrations up to 100 µg/mL and 40 mg/mL, respectively.

**Table 1:** Prediction of skin sensitization potency of MccJ25, pediocin PA-1, bactofencin A, nisin and reuterin.

Compounds	Concentration	RFI (CD86) (%)	RFI (CD54) (%)	
Microcin J25 (µg/mL)	100	9	− 18	Non-sensitizer
	50	14	− 32	
	6.25	− 55	− 114	
	0.05	− 131	− 208	
Pediocin PA-1 (µg/mL)	100	271	289	Sensitizer
	50	68	142	Non-sensitizer
	6.25	− 126	− 203	
	0.05	− 7	− 45	
Bactofencin A (µg/mL)	200	738	1216	Sensitizer
	100	76	311	
	50	72	66	Non-sensitizer
	1.56	127	139	
Nisin Z (µg/mL)	200	582	1284	Sensitizer
	100	16	576	
	50	− 41	48	Non-sensitizer
	1.56	98	145	
Reuterin (mg/mL)	40	0	0	Non-sensitizer
	20	0	0	
	1.25	0	0	
	0.63	126	126	
DNCB (µg/mL)	8	534	929	Positive control

### Skin irritation test

**Figure 5:**
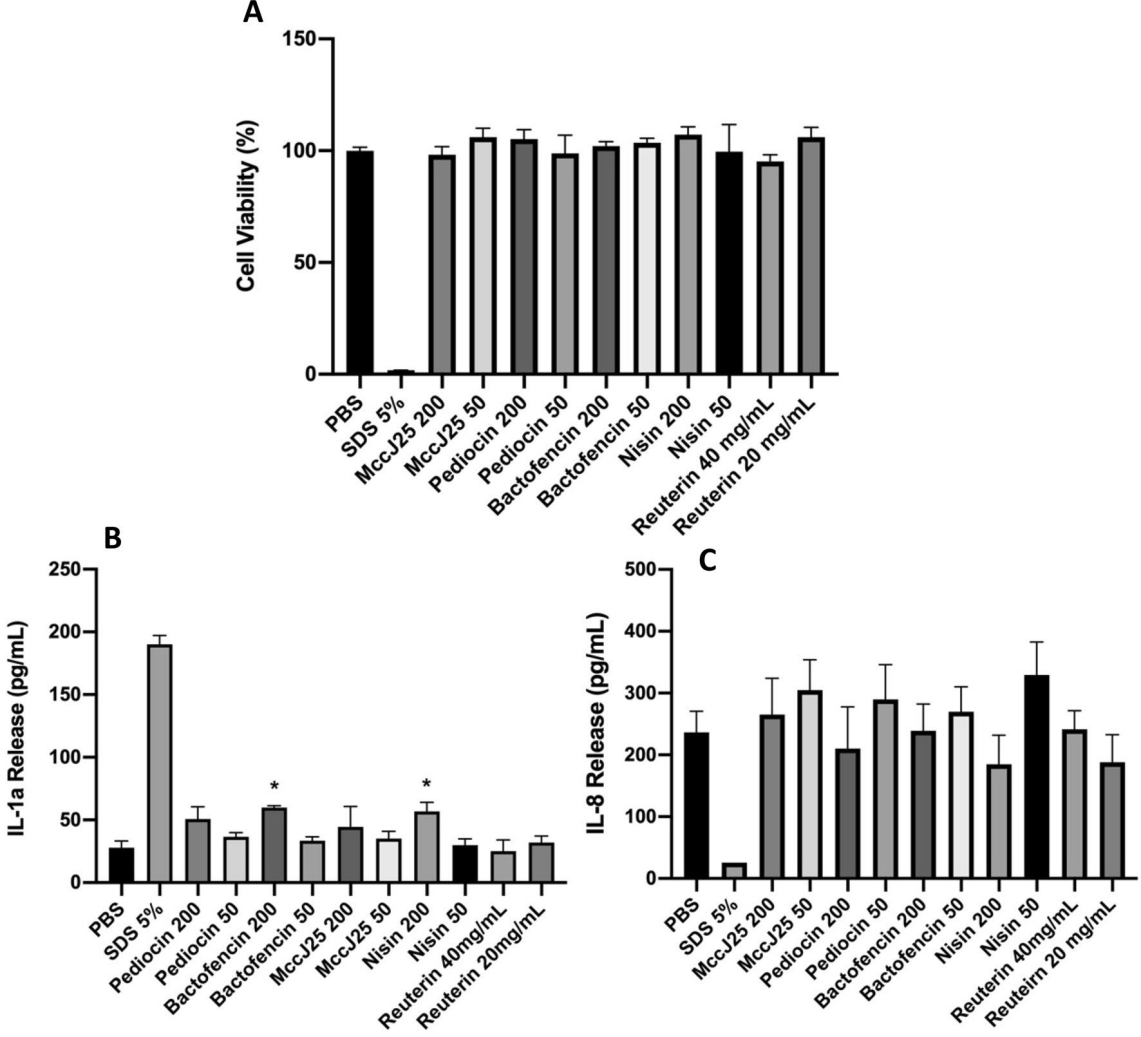
(**A**) Viability of the tissue (EpiDerm) after exposure to selected bacteriocins and reuterin. (**B**) IL-1α cytokine release. (**C**) IL-8 cytokine release. All data are represented as the mean ± SD for three replicates (n = 3). *P < 0.05 vs. control.

All the tested bacteriocins and reuterin did not affect tissue viability (Fig. 5A). All the tissues exposed to the compounds exhibited cell viabilities higher than 95% compared to the negative controls. Accordingly, bacteriocins did not cause skin irritation at the tested concentrations (200 µg/mL and 50 µg/mL for bacteriocins and 40 mg/mL and 20 mg/mL for reuterin). The positive control (5% SDS) reduced the cell viability to 1.8% and released 129 pg/mL IL-1α. Bacteriocins at a concentration of 200 µg/mL, slightly increased IL-1α release from the tissue into the basal medium. We obtained 50.6 pg/mL, 59.8 pg/mL, 44.6 pg/mL, and 56.8 pg/mL of IL-1α with pediocin PA-1, bactofencin A, microcin J25 and nisin Z, respectively (Fig. 5B). However, at 50 µg/mL, the release of IL-1α by the bacteriocins was slightly lower than that of the control (27.7 pg/mL). After comparisons using a one-way ANOVA followed by Tukey’s post hoc analysis, *p*-value < 0.001 for bactofencin A and nisin Z at 200 µg/mL compared to the negative control, suggesting that IL-1α release by these two bacteriocins was significantly different from the negative control. Released IL-8 levels induced by bacteriocins at 200 µg/mL were similar to those induced by the control (236 pg/mL), except for nisin Z, which showed a lower IL-8 release (185 pg/mL) (Fig. 5C). Therefore, for all compounds, the increase in IL-8 was statistically insignificant.

**Figure 6:**
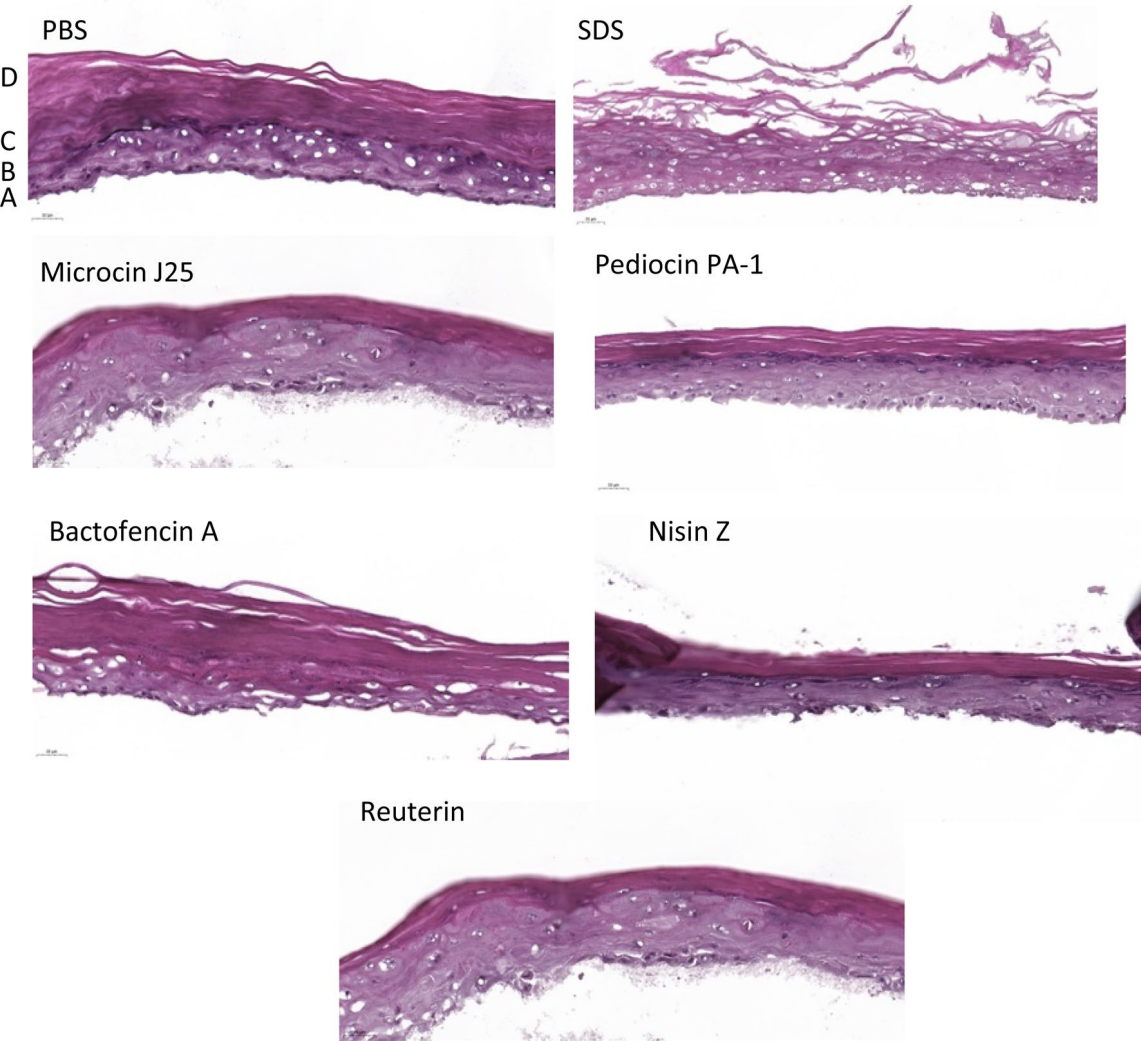
Histological analysis of skin tissue after exposing them to 200 µg/mL microcin J25, pediocin PA-1, bactofencin A, and nisin Z and 40 mg/mL reuterin. (**A**) Stratum basal membrane, (**B**) stratum spinosum, (**C**) stratum granulosum, (**D**) stratum corneum.

Finally, histological analysis was performed on the treated skin tissues to evaluate the impact of different antimicrobial compounds on skin integrity (Fig. 6). Exposing the skin tissue to PBS (used as a negative control) maintained intact skin morphology and well-differentiated layers stratum basal, spinosum, granulosum, and corneum. However, when the skin tissue was exposed to SDS (used as a positive control), significant disintegration of the skin morphology was observed at the corneum and granulosum levels. Bacteriocins and reuterin exposure left the skin tissue undamaged with intact morphology even at the highest tested concentrations (200 µg/mL and 40 mg/mL for bacteriocins and reuterin, respectively). A weak effect was observed with bactofencin A at the stratum corneum. However, this effect was similar to that observed with PBS; therefore, it was considered insignificant.

## Discussion

Several bacterial species produce antimicrobial compounds, including organic acids, proteins, bacteriocins and low-molecular-weight molecules, such as reuterin. bacteriocins have received increasing attention as antimicrobial compounds initially for food applications and more recently, for medical and veterinary sectors^25^. They are natural, with specific and narrow spectrum of activity, and high potency at nanomolar concentrations. However, the large-scale application of bacteriocins remains limited. Nisin is the only bacteriocin legally approved by the FDA in the United States and the European Food Safety Authority (ESFA) in Europe^26^. There is lack of data regarding the safety and toxicity of other bacteriocins that can be used as commercial antibacterial, hindering their industrial application.

The use of antimicrobial sanitizers (ABHS and NABHS) has increased considerably over the past two decades^27^. However, it has been reported that topical application of alcohol can cause irritation or contact dermatitis^5^. According to the FDA, vapor from alcohol-based sanitizers may have side effects such as headache, nausea and dizziness (FDA, 2020). Therefore, there is growing demand for natural alternatives to commercial sanitizers. Bacteriocins and reuterin as natural antimicrobial agents, are potential candidates that can be used in sanitizers. Their antimicrobial properties have been well studied, while limited data is available on their safety and toxicity. Since bacteriocins have not been studied for their use in hand sanitizers, there is no toxicity assessment available for their topical application. However, we need to identify potential health hazards of substances depending on the intended use and exposure route.

Therefore, we investigated, the toxicity and irritability of bacteriocins and reuterin for their use in sanitizers in the food, veterinary, and clinical settings. The toxicity of a substance varies depending on its exposure route and the tissue it interacts with. Although the primary route of exposure to sanitizers is dermal, there have been reports about their ingestion, especially in children^28^. Hence, in order to ensure the safety of bacteriocins and reuterin in sanitizers, all potential exposure routes must be assessed.

The cell culture method is a useful first-round screening tool that can also be used to determine the toxicity mechanism. Thus, we assessed the toxicity of the selected bacteriocins and reuterin on NHEK cells by measuring cell viability (neutral red assay) and membrane integrity (LDH assay). Bacteriocins and reuterin at concentrations up to 400 µg/mL and 80 mg/mL, respectively, did not reduce the relative viability and membrane integrity of NHEK cells. There was a dose-dependent increase in the number of viable cells for bactofencin A (at > 50 µg/mL) and reuterin (at > 2.5 mg/mL) that might correspond to cellular proliferation. However, caution should be taken when interpreting these results and further studies are required to evaluate the proliferative effect and the mode of action. A few studies on the oral toxicity of bacteriocins and reuterin have shown that they are non-toxic to mammalian cells at their effective concentrations close to the minimum inhibitory concentration (MIC) and minimum bactericidal concentration (MBC)^22,29,30^. Previously, we evaluated the cytotoxicity of reuterin on human colon adenocarcinoma Caco-2 cells for possible use as a food additive^18^. Reuterin was non-toxic to Caco-2 cells and did not affect the cell viability and membrane integrity at concentrations up to 80 mg/mL. Similar results were obtained in this study with NHEK cells. The results in the current work on NHEK cells might be used for skin toxicity and dermal exposure by topical application.

Some ingredients, such as active substances, preservatives, and fragrances, may cause allergies. Therefore, it is essential to test them for any side effects. A skin sensitizer is a substance that produces an allergic reaction after repeated contact with the skin^31^. Indeed skin sensitization is a critical toxicology endpoint and a key step to identify hazards of a substance applied directly to the skin^32^. The skin sensitization potential of substances has traditionally been assessed using animal models. However, due to regulatory requirements and ethical concerns, alternative approaches to animal testing are actively being developed. The h-CLAT assay is an in vitro model that tests the potential sensitization by chemicals by evaluating the upregulation of CD54 and CD58 in THP-1 cells^24^. The h-CLAT assay gives 84% and 83% accuracy for the CD54 and CD86, respectively, compared with animal and human tests^32^. To the best of our knowledge, very few studies have investigated the potential skin sensitization of sanitizer ingredients. In current study, we tested five different concentrations of the selected bacteriocins and reuterin. It was found that microcin J25 and reuterin at concentrations up to 100 µg/mL and 40 mg/mL, respectively, did not induce any significant skin sensitization, while nisin Z, pediocin PA-1, and bactofencin A showed sensitization potential at concentrations higher than 100 µg/mL. However, these concentrations are significantly higher than their MICs. Similarly Cebrián et al*.*^22^ found that bacteriocin AS-48 at a concentration up to 20 µg/mouse did not cause sensitization nor allergies in vivo using murine local lymph node assay (LLNA:BrdU-ELISA). Considering the similar nature of bacteriocins, they are expected to be non-sensitizers at similar concentrations. Voller et al*.*^33^ investigated the allergenicity of the ingredients of the most known brands of hand sanitizers. They showed that the five top allergens were tocopherol (51.3%), fragrance (40.0%), propylene glycol (27.5%), benzoates (25.0%), and acetyl stearyl alcohol (12.5%). Yang et al*.*^34^ reported that triclosan and polyhexamethylene guanidine (biocide) are skin sensitizers using the h-CLAT assay. As most antiseptics have similar ingredients, hence this assay is very important and could provides insights into the skin sensitization potential of similar antiseptic compounds.

Skin irritation is the “production of reversible skin injury following the application of a test compound for up to 4 h”. Until recently, the rabbit Draize test was used to assess the skin irritation potential of products. However, due to ethical and scientific limitations, the 3D in vitro EpiSkin model has been developed and validated by the European Centre for the Validation of Alternative Methods (ECVAM) as an alternative to animal tests. The EpiDerm assay measures cell viability as the endpoint. Our results showed that selected bacteriocins and reuterin did not affect cell viability at concentrations up to 200 μg/mL and 40 mg/mL, respectively, indicating that they are non-irritant and safe for the skin. The cytokine release assay showed similar levels of IL-1α and IL-8 in both the test and control tissues, indicating that the cells were undamaged after exposure to bacteriocins. However bactofencin A and nisin Z at 200 µg/mL showed a slight increase in IL-1α compared to control indicating that at concentrations higher than 200 µg/mL they may affect skin tissue; however it requires further studies. Histological analysis confirmed the absence of any sign of skin damage for 200 µg/mL of selected bacteriocins and 40 mg/mL of reuterin. Altogether, viability, cytokine production, and skin histology confirmed that all antimicrobial compounds tested in this study did not induce any undesirable side effects on the skin tissue.

In conclusion, the potential application of bacteriocins and reuterin in different formulations of sanitizers or disinfectants requires accurate toxicity data. In this study, different in vitro assays were used to assess bacteriocins and reuterin toxicity for topical applications. We generated crucial scientific data on the dermal toxicity of some bacteriocin (microcin J25, pediocin PA-1, bactofencin A, nisin Z) and reuterin. We showed that these bacteriocins and reuterin, could be safely used at concentrations close to their effective concentrations (MICs and MBCs). To use bacteriocins and reuterin in sanitizers formulations, their efficacy against skin pathogens such as S. aureus is required to be determined. In addition the effect of these molecules on normal skin microbiota should be evaluated using recent metagenomic approaches.

## Methodology

### Bacterial strains and culture condition

Microcin J25 was produced by *E. coli* MC4100 PTUC 202, provided by Prof. Sylvie Rebuffat (Muséum national d’Histoire naturelle, MCAM laboratory, Paris, France). It was cultured in Luria–Bertani (LB) (Difco Laboratories, Spark, MD, USA) at 37 °C. Reuterin was produced by *Lactobacillus reuteri* ATCC 53608 (STELA Collection, Laval University), it was cultured in Man-rugosa-sharpes (MRS) (Oxoid, Nepean, ON, Canada) under anaerobic condition (10% H_2_, 10% CO_2_, 80% N_2_) in Forma Anaerobic Chamber (Thermo Scientific, Waltman, MA, USA) at 37 °C.

Antimicrobial activity of the pure compounds was evaluated using Gram-positive and Gram-negative indicator strains. *Salmonella enterica* subsp. *enterica* serovar Newport ATCC 6962 (later referred to as *S.* Newport, ATCC) (STELA Collection, Laval University) was used an indicator strain for microcin J25 and reuterin. It was cultured in LB at 37 °C. *L. ivanovii* HPB28 (Canada Health Protection Branch) was used as an indicator strain for pediocin PA-1 and nisin Z. It was cultured in Tryptone Soy Broth TSB enriched with 0.6% yeast extract at 30 °C. *Staphylococcus aureus* ATCC 6538 (STELA Collection, Laval University) was used to test bactofencin A activity, and it was cultured in TSB at 37 °C. All the strains were stored frozen in relevant media supplemented with 20% (vol/vol) glycerol in 20% at − 80 °C.

### Production and purification of bacteriocins and reuterin

*Microcin J25* was obtained from *Escherichia coli* MC400 PTUC202 cultured in minimal medium (M63) in previously established conditions^35^. Microcin J25 was first purified from the culture supernatant by solid-phase extraction using a Sep-Pak® C18 35 cc cartridge (Waters™, Milford, MA) and then further purified to homogeneity (up to 95% purity) using RP-HPLC (Beckman Coulter System Gold Preparative HPLC system, Mississauga, ON, Canada) on a preparative C18 column (Luna 10 µm, 250 mm × 21.10 mm, Phenomenex, CA, USA). Pure microcin J25 was quantified using an analytical HPLC system (Waters, Milford, MA) equipped with an analytical C18 column (Aeris 3.6 µm PEPTIDE XB-C18, 250 × 4.6 mm, Phenomenex, CA USA) according to Ref.^35^.

*Pediocin PA-1* and *Bactofencin A* were prepared using standard solid-phase peptide synthesis (SPSS) using the Fmoc/tBu strategy according to a previously established protocol^36,37^. Synthetized linear analogs of pediocin PA-1 (M31L) and bactofencin A (M14L, M18L) showed similar activity to wild types while their stability was improved. Pediocin PA-1 and bactofencin A were purified to > 95% homogeneity by RP-HPLC (Prominence HPLC, Shimadzu) on a Kinetex EVO C18 column (250 mm × 21.2 mm, 300 Å, 5 μm) (Prominence, CA, USA) using ultraviolet detection at 220 nm and 254 nm.

*Nisin Z* was purified from Niseen®-S, a commercial nisin preparation (Fromagex, Quebec, Canada). Nisin was purified using the salting-out method according to a previously described protocol (Gough et al. 2017) and quantified using an analytical HPLC system (Waters™, Milford, MA) equipped with an analytical C18 column (Aeris 3.6 µm PEPTIDE XB-C18, 250 × 4.6 mm, Phenomenex, CA USA).

*Reuterin* was produced by *L. reuteri* ATCC 53,608 as described previously^14^. An overnight grown culture of *L. reuteri* in MRS media supplemented with 20 mM glycerol was centrifuged (1500×*g*, 10 min, 20 °C), and washed with 0.1 M potassium phosphate twice before resuspending them in 300 mM glycerol and incubating anaerobically for 45 min at room temperature. After two rounds of centrifugation, the supernatant containing reuterin was filtered, and the purity and quantity of reuterin were determined using an analytical HPLC system (Waters™, Milford, MA) equipped with an ICsep-ion-300 column (Transgenomic, San Jose, CA) as previously described elsewhere^18^.

### Inhibitory activity by agar well diffusion assay

Antibacterial activity of the tested compounds was determined using an agar well diffusion assay as described previously^38^. Briefly, 80 µL of bacteriocins (200 µg/mL) and reuterin (20 mg/mL) were placed in wells in their respective media seeded with bacterial strains (at concentration of 1%) in soft agar (0.75% agar). After overnight incubation at appropriate temperatures, the zone of inhibition for each compound was measured.

### Cell culture

The normal human epithelial keratinocytes (NHEK) cells were purchased from Lonza (Lonza, Walkersville, MD, Lot #0000665959). Cells were cultured in keratinocyte growth medium as per the manufacturer’s instructions (KGM, Bulletkit, Lonza, Basel, Switzerland) in a humidified atmosphere containing 5% CO_2_ at 37 °C. Cells were passaged every 2–3 days and maintained at 80% confluency. They were used for cytotoxicity assays within 4 passages.

The human leukemia cell line THP-1 (TIB-202) ATCC (Manassas, VA, USA) was used for the h-CLAT assay. Cells were cultured in a humidified atmosphere containing 5% CO_2_ at 37 °C in RPMI-1640 medium supplemented with 10% fetal bovine serum (FBS), 0.05 mM 2-mercaptoethanol, 100 units/mL penicillin 14, and 100 µg/mL streptomycin. The cells were maintained at a density between 8 × 10^5^ and 1 × 10^6^ cells, and the medium was changed every 2–3 days.

### Cytotoxicity assay

Neutral red assay: The viability of NHEK cells was evaluated using the neutral red assay according to Ref.^39^. First, NHEK cells were seeded at 7500 cells/well in a 96-well plate and allowed to settle for 48 h. Then, 100 µL of serial dilutions of bacteriocins were added to the cells to obtain final concentrations of 0.39, 0.78, 1.56, 3.125, 6.25, 12.5, 25, 50,100, 200, and 400 µg/mL. For reuterin, final concentrations of 0.078, 0.156, 0.3125, 0.625, 1.25, 2.5, 5, 10, 20, 40, 80 mg/mL were used. The cells were incubated at 37 °C with 5% CO2 for 48 h. The medium was then aspirated, and cells were incubated for 3 h with neutral-red solution (33 µg/mL in EMEM). To solubilize the incorporated dye, 100 μL of fixative (50% ethanol and acetic acid) was added, and the plate was incubated for 10 min with constant rocking. The absorbance was measured at 540 nm using a spectrophotometer (SPARK® 20 M, Tecan), and the cell viability was calculated by dividing the absorbance of the treated cells by that of the untreated cells (% viability).

LDH release assay*:* The CytoTox-ONE™ (Promega, USA), was used to evaluate bacteriocin cytotoxicity via LDH release under conditions similar to that for the neutral red assay. After exposing the cell cultures to different bacteriocins and reuterin concentrations, 100 μL of their supernatant was transferred to a new 96-well plate, and 100 μL of CytoTox-ONE™ reagent was added in each well. After 10 min of incubation, the reaction was terminated by adding 50 μL of stop solution. Absorbance was measured using a fluorescence spectrophotometer at an excitation wavelength of 560 nm and an emission wavelength of 590 nm. Compound-treated values were blanked against the reading obtained from control-treated cells and were calculated as a percentage of maximal LDH release (lysis buffer treated cells) as follows:

$$\text{\% Cytotoxicity}=\left(\frac{\left(\text{Compound exposure LDH activity}\right)- \left(\text{culture medium background}\right)}{\left(\text{Maximum LDH activity }-\text{ culture medium background}\right)}\right)\times 100.$$

### Skin sensitization assay (h-CLAT)

The human cell line activation test (h-CLAT) is an in vitro assay based on dendritic cell (DC) activation as it is one of the main steps in skin sensitization. Dendritic cells upregulate the expression of CD86 and CD54 cell surface proteins upon exposure to sensitizers. Cells surface markers CD54 and CD86 were upregulated according to OECD guidelines^24^. THP-1 cells were seeded at a density of 1 × 10^6^ cells/mL in 96-well plates. After 24 h, cells were exposed to different concentrations of each compound: Microcin J25 100, 50, 6.25, 0.05 µg/mL; pediocin PA-1 100, 50, 6.25, 0.39 µg/mL; nisin 200, 100, 12.5, 6.25 µg/mL; bactofencin A 200, 100, 50, 1.56 µg/mL; and reuterin 40, 20, 1.25, 0.63 mg/mL and incubated for 24 h at 37 °C in 5% CO_2_. After incubation, the cells were centrifuged and washed twice with staining buffer (PBS containing 1% bovine serum albumin). To block the Fc receptors, cells were incubated with 0.05% of human IgG antibodies (Sigma-Aldrich) for 10 min at 4 °C. The cells were then stained with FITC-labelled anti-human CD86 antibody (BD Biosciences) and PE anti-human CD54 antibody (BD Biosciences) at 4 °C for 30 min. Following washing the cells with staining buffer, the expression of the cell surface antigens was analyzed using flow cytometry (Fortessa™ X-20, Becton Dickinson, San Jose, CA, USA), and FlowJo™ software v 10.7 (Ashland, OR) was used for the analyses. Dead cells were excluded using 7-aminoactinomycin D (7-AAD). FITC- or PE-IgG1 isotype controls (BD Biosciences) were used to determine the basal fluorescence signals. Dinitrochlorobenze (DNBC) (Sigma-Aldrich, ST. Louis) was used as a positive control.

Based on the geometric mean fluorescence intensity (MFI), the relative fluorescence intensity (RFI) of CD86 and CD54 for positive control (ctrl) cells and chemical-treated cells were calculated according to the following equation:

$$RFI = \left(\frac{MFI\, of \,chemical{\text{-}}treated\, cells-MFI\, chemical{\text{-}}treated\, isotype\, control}{MFI\, of \,vehicle{\text{-}}treated \,cells-MFI chemical{\text{-}}vehicle\, isotype \,control} \right)\times 100.$$

### Skin irritability assay (epidermal model)

The reconstructed in vitro model human epidermis tissues EpiDerm™, EPI-200 were purchased from MatTek Corporation (Ashland, MA, USA). This model consists of fully differentiated, three-dimensional normal, human-derived epidermal tissue grown at the air–liquid interface on a semi-permeable tissue culture insert. In addition to skin irritation this model can be used in different applications such as skin percutaneous absorption/permeation testing; skin genotoxicity screening; skin metabolism; chemical warfare agents; and cutaneous microbiology/infection, etc.^40^. The experiment was carried out according to the manufacturer’s protocol^41^ and OECD Test Guideline 439^23^. The 3D structure of Epiderm™ consists of well-differentiated layers basal, spinous, and granular, and the cornified epidermal layers. The kit was received within 2 days and the inserts containing tissues (surface area 0.6 cm^2^) was transferred to a 6-well plate filled with pre-warmed maintenance media immediately upon arrival and it was incubated at 37 °C under 5% CO_2_ in a humidified atmosphere.

Tissues were topically treated with 40 µL of bacteriocins (50 and 200 µg/mL) and reuterin (20 and 40 mg/mL). 5% SDD was used as positive control and PBS as a negative control. After 2 h of incubation, the tissue and medium were collected for analysis.

The viability of the tissues was assessed by the MTT assay. After exposing the tissues to compounds, they were removed from the inserts, rinsed with PBS, placed in a 24-well plate containing 300 µL MTT solution, and incubated for 3 h at 37 °C and 5% CO_2_. After incubation, the tissue inserts were placed in a fresh 24-well plate (extraction plate), immersed in 2 mL of extractant solution, and sealed, and put on an orbital shaker for 2 h at room temperature. Two aliquots per tissue were collected and placed in 96-well plates, and the optical density was recorded at 570 nm. The viability (%) was calculated for each tissue, as follows:

$$\% Viability = \left(\frac{OD\, sample}{OD \,negative\, control}\right) \times 100.$$

The concentrations of the cytokines IL-α1 and IL-8 released into the tissue culture medium, at the end of the exposure time (24 h) were measured by commercial immunoassays (R&D Systems). Each sample was evaluated in triplicate.

Histological analysis was performed on the tissues after exposure. Tissues were fixed with 4% paraformaldehyde for 1 h followed by an overnight incubation in PBS containing 20% sucrose and 0.05% sodium azide. Tissues were embedded in OCT and sectioned for hematoxylin and eosin staining. Slides were examined using a Pannoramic Midi slide imaging system (3D Histech), and images were analyzed using CaseViewer™ v.4.2.

### Statistical analysis

The results are expressed as the mean ± Standard error of at least three independent experiments. Dose–response curves and bar charts were created using GraphPad Prism v.8.2 (GraphPad Software, San Diego, CA, USA). Means were compared using one-way analysis of variance (ANOVA) followed by Tukey’s test (GraphPad Prism version 8.2). *p* < 0.05 was considered statistically significant.
